# Psychological Morbidities Associated With Excessive Usage of Smartphones Among Adolescents and Young Adults: A Review

**DOI:** 10.7759/cureus.30756

**Published:** 2022-10-27

**Authors:** Ashwini S Rathod, Abhishek Ingole, Abhay Gaidhane, Sonali G Choudhari

**Affiliations:** 1 Department of Epidemiology and Public Health, School of Epidemiology and Public Health, Jawaharlal Nehru Medical College, Datta Meghe Institute of Medical Sciences, Wardha, IND; 2 Department of Community Medicine, School of Epidemiology and Public Health, Jawaharlal Nehru Medical College, Datta Meghe Institute of Medical Sciences, Wardha, IND

**Keywords:** excessive use, smartphone, psychological morbidity, young adults, adolescents

## Abstract

Adolescents and young adults have progressively become smartphone and internet-dependent. Its addiction is affecting them mentally and physically. Due to its overutilization, it is causing a detrimental effect on them. These dilemmas need to be acknowledged. Furthermore, the goal is to reduce overutilization and overreliance, and the task is to reduce the heavy toll on their mental and psychological condition. We need to review the evidence linking smartphone and social media use with psychological morbidities among adolescents and young adults. The aim of this study is to summarize the present situation and the correlation between smartphones and mental health. Cognitive, emotional difficulties, poor cognitive function, impulsivity, shyness, social networking addiction, low self-esteem, and some medical issues like insomnia, anxiety, depression, and a lack of cognitive control have been linked to excessive smartphone use.

## Introduction and background

Smartphone

The 21st century is referred to as the era of information technology. The astonishing technologies of wireless communication and the internet have caused radical shifts in the communication industry [1]. The first smartphone was released in 2007 [1]. Since then, smartphones have become a necessity for daily life in all societies [2]. Smartphone use and ownership have skyrocketed significantly during the last ten years. For instance, there were around 2.1 billion smartphone users worldwide in 2017, and it was predicted by World Health Organization (WHO) that the number would approach 7.26 billion by 2022 [2]. After China, India has the second-highest smartphone user base [3].

The popularity of mobile communication and computing is now on the rise. There are several types of mobile networking systems available in the market, including 3G, 4G, 5G, Bluetooth, Wi-Fi, and Wi-Max technologies, as well as the widespread use of mobile access control systems such as laptops, tablets, and smartphones [4].

Given the frequency of smartphone use among Indian youths, most young people from lower socioeconomic categories throughout the world are vulnerable to the effects of readily available and less expensive smartphones. Some studies on smartphone use and its consequences have revealed that teens under the age of 15 years are most affected in India and throughout the world. For addiction control, issues require excellent investigation, emphasizing technology's role in developing fantasies and acting out behaviors [4].

Adolescents and young adults 

According to the WHO, adolescents are between 10 and 19 years of age, whereas young adults are between 15 and 24 years of age [5]. The salient attributes of adolescents and young adults are emotional, psychological, physical, and social development. They have rapid but uneven physical growth and experience sexual maturity and the onset of sexual behavior. The desire for experimentation and exploration is insurmountable. Development of adult mental process, the transition from dependence to relative independence and self-identity takes away years of their life [4]. 

They are more risk-taking and sensation-seeking. They are more acceptive, conscientious, impulsive, extra-socially oriented, less agreeable, and more able to inhibit behavior [5]. They also derive a more significant portion of their happiness and life satisfaction from their peers. Smartphone addiction may severely affect this self-development process and sometimes may hamper their growth. This needs correction and major interventions [4].

Smartphone impact on adolescents and young adults

Globally, there is an appreciable argument on addiction and excessive use of smartphones among adolescents and its subsequent impact on their health. Smartphone addiction can damage not only interpersonal skills but also cause major health concerns and have a severe psychological impact on teenagers [4]. Smartphones have gained popularity among adolescents and young adults due to the tendency to choose the virtual world, which is more entertaining than being with their friends and parents. For social interaction, social networking services (SNS) like messenger, email, Facebook, WhatsApp, Twitter, Snapchat, etc. [6]. Browsing websites and apps like YouTube, Google, Yahoo, porn websites, etc. are also used frequently. Also, shopping portals, reel apps, and gaming apps are more commonly used by a vast number of smartphone users, who are majorly adolescents and young adults [4].

Smartphone addiction has caused dysfunctional impulsivity to regularly check mobile phones. Average people spend about 5 hours a day on their phones [7]. More time is spent than eating, exercising, and socializing with friends. Common indicators include maintaining their social media persona, responding to messages quickly, being active on social media, using headphones frequently, and having sleeping issues [4,8,9].

Adolescence plays an important role in grooming a person, making them capable enough to adapt to their surroundings. Smartphone usage primarily impacted adolescents and young adults by predisposing them to mental health issues, insomnia, cyberbullying, anxiety, depression, obesity, false prestige, self-control issues, physiological stress, vision problems, mind-wandering, attention deficit-hyperactivity disorder (ADHD), and obsessive-compulsive disorder (OCD). Smartphone addiction decreases life satisfaction and increases the chances of potential health risks and negative thinking styles in adolescents [10-12].

Addiction

Addiction is the inability to stop using a substance or engaging in an activity, even knowing it has a negative impact on physical and psychological well-being. And some signs suggest that someone may have an unhealthy dependence on a smartphone, like the need to drop everything and check the phone every couple of minutes. Due to this, productivity at work is damaged because of regularly stopping to check the phone, and sleep is disturbed due to continuously waking up to check the phone [1,3,4,10].

## Review

**Figure 1:**
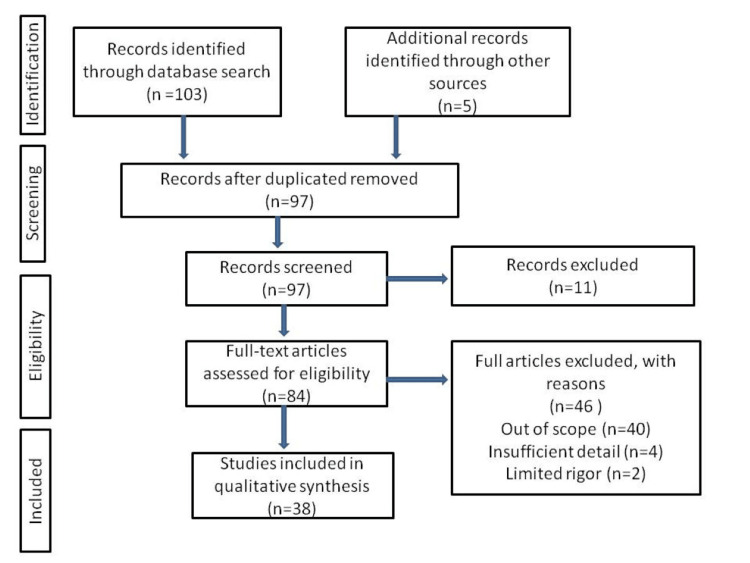
Inclusion and exclusion criteria of this study. The image is created by the author (ASR) of this study.

Web of Science search, PubMed Central, and Google Scholar search engines utilized the terms "smartphone addiction" and "excessive smartphone use," yielding 84 research studies in English. Figure 1 shows the inclusion and exclusion criteria.

Smartphone addiction and its detrimental effects 

Negative Psychological Effects

Negative attitudes and feelings of fear due to smartphone usage are related to an increased risk of depression and anxiety [13]. According to Jones, who carried out a study among students of Elon University, North Carolina, and the United States, students are always hooked to their mobile phones, and that determines their behavior and negative psychological effect [14].

Stress

Stress is any type of change that causes psychological, emotional, or physical strain. In one study, young German internet users were questioned, and it was discovered that communication load was associated with emotions of stress and indirectly influenced grief and anxiety [12]. Another study reached similar conclusions, a questionnaire was distributed to 439 students aged 12-17 years, including parents and young adults from Central Switzerland, and it was discovered that smartphone usage during the night hours was common among adolescents and that being awake all night resulted in poor perceived health [13]. There was no tangible link between memory performance and mobile phones. As a result, it was found that digital stress is a big stimulant and causes major psychological health effects [13].

Depression 

Depression is a psychological disorder that results in persistent emotions of sadness, loneliness, and lack of joy and is thought to be highly correlated with addiction to smartphone usage. The majority of studies on this issue revealed that there is a relationship between these two variables, that is, depression and smartphone usage [14]. Brian, in 2013 conducted research titled "Two Days Without Phone," and she conducted her research on her students, Kenny and Franchesca. She found that while Franchesca was comfortable not to have her phone nearby and easily handed over her phone, Kenny did not want to lose his [15]. In 2012, researchers discovered a significant increase in smartphone usage among teens and symptoms of an increase in depression which ultimately lead to suicidal thoughts [15].

Anxiety and Sleep Loss Pattern 

Anxiety is a type of mental illness that causes fear, nervousness, worry, and apprehension [12]. In 2017, Boumosleh and Jaalouk studied whether depression and anxiety led to smartphone habits. Their cross-sectional study showed that sadness and anxiety are good predictors of smartphone addiction [12]. A descriptive study conducted by Fisoun et al. found that internet addiction is similar to drug addiction. If internet addiction persists, it will end in the same outcomes as alcohol addiction [16]. Another observational research suggested that sleeplessness can lead to depression. The subjective experience of finding it difficult to fall asleep or stay asleep is known as insomnia or sleeplessness [11]. Li et al. in 2016 conducted a prospective cohort study and hypothesized that sleep deprivation and the risk of depression are related [17]. Gutiérrez et al. discovered that problematic smartphone usage was linked to sleep deprivation, despair, and anxiety [11]. Another Japanese study involving 94,777 young adults found that using a smartphone to send messages and calls after lights out was connected with sleep disturbances such as poor sleep quality, short sleep duration, insomniac symptoms, and excessive daytime sleepiness [18].

Cyberbullying

Cox Communications carried out a study on students aged 13-18 years, of which 15% of students had experienced cyberbullying online, 10% had experienced it on mobile phones, and 5% were engaged in cyberbullying against another person using a mobile phone according to a poll, cyberbullying cause teens to become depressed and resent going to school [12]. Additionally, this research has shown that children who experience cyberbullying are more likely to experience psychosomatic issues, such as chronic headaches, sleep issues, anxiety, and despair [19]. Cyberbullying is more challenging to spot because it goes unnoticed than physical bullying. Because the internet provides anonymity, criminals can remain undetected [11].

False Prestige

Adolescents may now easily access any of the most recent advancements in mobile technology. Adolescents who are naive for their age may accept the majority of things available on social media, can become serious about such things, and can get influenced, even if they may not be accurate. They might create a false sense of status and start living in a dream world. Some people could turn to crime to live out their desires [11].

Obesity

Adolescents' obesity may result from constant mobile phone usage. Harvard T.H. Chan School found that adolescents who use their phones for many hours daily are more likely to gain weight. According to the study, adolescents who spend more than five hours a day in front of a device are 43% more likely to receive less sleep and are less tend to do exercise, which can lead to obesity [20].

Vision Problems

As per recent reports, there are many cases of vision problems that are mainly associated with increased smartphone screen time. Symptoms are ocular muscle fatigue, redness, lacrimation, irritation, blurry vision, and dry eyes. In a research of 30 medical students, smartphone vision syndrome was discovered in 83% of the participants. Adolescents and young adults use smartphones excessively, which outweighs their value and leads to new issues. Even if it is not possible to stop adolescents from using a phone, however, specific limits can be set on how much time to spend on it [21]. 

Cognitive Behavior

Cognition is the mental activity or process of learning and comprehending things through experience, the senses, and thought [5]. Ellison in 2012 asked that “are smartphones making us dumber?” Morin in 2013 asked that “is your smartphone making you fat and lazy?” Greenfield in 2013 stated that reliance on smartphones and related technologies is not aiding mental functioning, but rather, is having a negative impact on our ability to think, remember, pay attention and regulate emotion [22]. Some have even made the claim that modern connectedness is “rewiring our brains” to constantly crave instant gratification and that this threat to our society is “almost as important as climate change” [22]. The authors interpreted this result as evidence that increased daily multitasking leads individuals to experience greater difficulty in recruiting cognitive control resources. Regular media multitaskers had less grey matter in their anterior cingulate cortex, according to research by Loh and Kanai in 2016 [23]. This suggests that this behavior may directly affect the structural characteristics of a critical brain region involved in attention regulation [23].

Loneliness, Self-Control, Worry, and Anger

Mahapatra in 2019 showed that sticking to smartphone use leads to a lack of self-regulation and loneliness which ultimately damages family relations, and leads to interpersonal conflicts and poor academic performances. High measures of worry and anger are seen in students with problematic smartphone use. Excessive reassurance-seeking behavior mediated the association between rumination and problematic smartphone use [24]. 

Physical Fitness

Addicts to smartphones were less likely to walk every day. In particular, smartphone addiction may have a severe impact on physical health by limiting the quantity of exercise, such as walking, leading to an increase in fat mass and a decrease in lean mass, both of which have negative health effects [25].

Migraine

It has been observed that smartphone usage leads to increased headache duration and frequency in migraine patients. In addition, as smartphone usage rises, it results in decrement in sleep quality and our quality of life. This is especially true for migraine sufferers who use their phones excessively, which is linked to poor sleep and daytime drowsiness [26].

Fear of Missing Out

Fear of missing out (FOMO) has been characterized as concern over missing good events [27]. It is also seen as a motivation for remaining informed of what others are doing on social media. It is such a common occurrence that a scale has been developed to quantify it. A significant association was found between FOMO scale scores and problematic smartphone use in a study of Turkish adolescents [27]. According to a regression study, there was up to 28% of variation in FOMO scale scores due to problematic smartphone use in research on 2663 Belgian teenagers. It was found that there was a problem with FOMO due to excessive use of private social media sites [28]. It predicted phubbing behavior is directly and indirectly linked with problematic smartphone usage, for example, spending too much time on Facebook [28].

Studies conducted all over the world about smartphone addiction among adolescents and young adults and its detrimental effects are mentioned in (Table 1) [6].

**Table 1:** Detrimental effects of smartphone addiction in adolescents and young adults The table is adapted from Wacks and Weinstein (2021) [6]. ADHD: attention deficit-hyperactivity disorder

Authors	Year	Population	Finding
Kim et al. [29]	2020	South Korea (350 children)	Reduced sleep quality as well as duration
Lemola et al. [30]	2015	362 adolescents sleep	Sleep disruption mediated the association between electronic media usage in bed before sleep and depressive symptoms
Tamura et al. [18]	2017	Japan (295 high school students aged 15-19 years)	Shorter sleep duration and insomnia
Liu et al. [31]	2019	Technical college in China (4733 students aged: 14-24 years)	Anxiety, depression, sleep disturbances
Farooq et al. [32]	2019	Pakistan (500 university students)	Poor sleep quality and disturbed sleep pattern
Dharmadhikari et al. [33]	2019	India (195 medical students)	Higher perceived stress and poor sleep quality
Demir and Sumer [26]	2019	123 migraine young adult patients smartphone	Smartphone use has increased the length and incidence of headaches. Its excessive usage was linked to poor sleep quality, daytime drowsiness, and a worse quality of life
Turgeman et al. [34]	2020	140 university students	Overuse of smartphones has been linked to social anxiety, anxiety, and depression
Darcin et al. [35]	2016	367 university students	Loneliness and social anxiety
Eichenberg et al. [36]	2019	Spanish (845 adolescents)	Maladaptive cognitive-emotion regulation was significant in problematic smartphone users
Kim et al. [10]	2019	4,512 middle- and high-school students excessive	Excessive smartphone use was predicted with ADHD
Kapkın et al. [37], Domoff et al. [38], Mahapatra [24]	2020, 2020 and 2019	Turkey (443 high-school students ) 193 adolescents 350 students (age: 15-20 years)	Excessive smartphone use was linked to childhood emotional abuse, emotion management issues, uncontrolled eating, constrained eating, food addiction, a more significant percent body fat, and loneliness

## Conclusions

Anything beyond what we need is poison. The same applies to the growing addiction to smartphone usage among adolescents and adults. It has become a public health issue leading to some concerning issues like anxiety, insomnia, over-dependency, behavioral and psychological changes, etc. Smartphone unquestionably improves access to knowledge and connectivity, but at the same time, its addiction is quite alarming. If guardians intend to give adolescents a phone, ensure they strictly supervise their phone usage. Otherwise, they may spend the entire day staring at a screen. If not, it will have irreparable consequences for the person's physical health, relationships, studies, friendships, and family bonds. They must also agree on smartphone usage guidelines. And thus, this paper tries to pinpoint prior research by examining the links between smartphones and mental health problems related to addiction and helping us justify whether or not it is making us antisocial and unhealthy.
